# Characterization and clinical management of abnormal cytology findings in pregnant women: a retrospective analysis

**DOI:** 10.1007/s00404-022-06699-7

**Published:** 2022-08-17

**Authors:** Rosa Freudenreich, Martin Weiss, Tobias Engler, Felix Neis, Melanie Henes

**Affiliations:** 1 Department of Women’s Health, University Hospital Tübingen, Calwerstraße 7, 72076 Tübingen, Germany; 2 Eberhard Karls University of Tübingen, Tübingen, Germany

**Keywords:** Pregnancy, Dysplasia, Cervical cancer

## Abstract

**Purpose:**

The diagnosis of cervical intraepithelial neoplasia during pregnancy poses a great challenge to the treating clinician and the patient. According to the current guidelines, watchful waiting during pregnancy can be justified. Only in cases of invasion, immediate treatment may be indicated. However, few data are available on the management of cervical dysplasia during pregnancy. Further research is important for counselling affected women.

**Methods:**

Data of pregnant patients with suspected cervical dysplasia who presented to the University Women’s Hospital Tübingen between 2008 and 2018 were evaluated retrospectively. Colposcopic, cytologic, and histologic assessment was performed for diagnosis. Data on remission, persistence and progression of disease based on histologic and cytologic assessment and the mode of delivery were correlated.

**Results:**

142 patients were enrolled. Cytology at first presentation was PAPIII (-p/-g) in 7.0%, PAPIIID (IIID1/IIID2) in 38.7%, PAPIVa (-p/-g) in 50.0%, PAPIVb (-p) in 2.8%, and PAPV (-p) in 1.4%. All cases with suspected invasion were recorded at the initial presentation. Complete histological or cytological remission was observed in 24.4%, partial remission in 10.4%, persistence in 56.3%, and progression in 8.9%. In two cases (1.5%) progression to squamous cell carcinoma occurred.

**Conclusions:**

Watchful waiting for cervical intraepithelial neoplasia during pregnancy seems to be sufficient and oncologically safe. It is important to exclude invasion during pregnancy, to perform frequent colposcopic, cytologic and histologic examinations and to ensure a postpartum follow-up examination to initiate the treatment of high-grade lesions. Spontaneous delivery seems to be safe in patients with cervical dysplasia, Caesarean section is not indicated.

## What does this study add to the clinical work

**Table:** 

A watchful waiting strategy in patients with diagnosed cervical intraepithelial neoplasia during pregnancy seems to be sufficient and oncologically safe. Invasive carcinoma should be excluded and frequent colposcopic, cytologic and, if necessary, histological examinations as well as a postpartum follow-up examination should be ensured. Spontaneous delivery seems to be safe in patients with cervical dysplasia.	

## Introduction

The management of pregnant women with abnormal cytological findings is a challenge for the attending physicians. On the one hand, maternal health must be preserved, and invasive lesions must be excluded; on the other hand, the unborn child should not be exposed to any avoidable risks. Cervical intraepithelial neoplasia (CIN) is particularly common in women aged 20–34 years [1, 2]. The average age of a woman at childbirth was 31.3 years in Germany in 2018 [3].

In pregnancy, incidences of CIN are reported to be between 1.3 per 1000 [4] and 7 per 1000 pregnancies [5], for cervical carcinoma 3.3 per 100.000 births [4]. For abnormal PAP smears (PAPII-p +), incidences range from 1.4% [5] to 5% [6].

CIN 1 and CIN 2 have a high spontaneous regression rate, CIN 3 shows progression in 12% of cases [7]. Persistent infections with high-risk human papillomaviruses (HPV) play an important role in the development of cervical dysplasia and cervical carcinoma [8, 9]. It is important that 80–90% of all HPV infections resolve spontaneously [10, 11].

The management of abnormal cytology findings in pregnant women is almost the same as recommended in non-pregnant women. Endocervical curettage is contraindicated in all cases [12]. Pregnancy-associated changes of the cervix may complicate the interpretation of Pap smears [6, 13]. Nevertheless, Pap smears during pregnancy are an important and reliable diagnostic tool [14].

Colposcopy is an important tool for evaluating cervical dysplasia in pregnancy. The transformation zone is usually visible in pregnancy. Enlargement of the cervix and local oedema may occur during pregnancy due to hormonal influences. Inflammation as well as increased vascularization and high vulnerability of the epithelium may complicate the differentiation from invasive processes [15, 16]. There are reports of tendencies to overestimate colposcopic findings in pregnant women [17]; therefore, colposcopy should always be performed by experienced clinicians [12, 18]. In cases of suspected high-grade cervical dysplasia, colposcopically guided biopsies should preferably be taken in the 16–20th week of gestation. Outside this period, biopsies may be performed to exclude invasive lesions after an assessment of risks [19, 20]. The primary goal during pregnancy is to exclude invasive carcinomas. The preferred method for the treatment of cervical dysplasia is the loop electrosurgical excisional procedure (LEEP) [12]. However, the only indication for excision in pregnancy is a reasonable suspicion of the presence of an invasive lesion. The procedure can be associated with a high risk of complications when performed during pregnancy [5, 21]. There is a lack of data concerning the management of CIN in pregnant women. Studies available are mostly retrospective analyses with rather small group sizes. Therefore, further research on the evolution and management of cervical dysplasia during pregnancy is important.

The aim of this work is to further assess the course of dysplasia during pregnancy, to generate data especially on remission, persistence, and progression of CIN and to facilitate the counselling of affected women. In addition, diagnostics in pregnancy and postpartum as well as possible effects of the mode of delivery on the probability of remission will be investigated.

## Methods

### Study design

This is a retrospective, monocentric data analysis. Ethics approval was obtained by the ethics review board of the University Hospital Tübingen. In accordance with the ethics approval, obtaining informed consent from the patients was not required. All data were analysed anonymously. The data were taken retrospectively from the medical record; there was no patient contact during the study.

### Patient sampling and clinical information

Pregnant patients who presented to the dysplasia clinic at the University Women’s Hospital Tübingen with suspected cervical dysplasia between January 2008 and December 2018 were included in the study. Data on general medical history and current pregnancy, cytologic, colposcopic, and histologic findings, as well as information on HPV status and therapies performed were obtained from the medical record. In case of abnormal colposcopy and cytology, colposcopy-guided biopsies were taken preferably in the 16–20th week of pregnancy. Endocervical curettage was never performed. Usually, follow-up appointments with repeated Pap smear and colposcopy were made every 8–10 weeks. Further biopsies were taken only in cases of suspected invasion. Postpartum consultation took place 6–8 weeks after delivery. Classification of cytologic findings was performed according to Munich Nomenclature II [22] until June 2014 and Munich Nomenclature III [23] from July 2014. Pap smears were performed under colposcopic view using a cytobrush. External cytological findings were taken from the medical record. To standardize the different classifications, the findings were grouped as follows: PAPIII (PAPIII, PAPIII-g, PAPIII-p); PAPIIID (PAPIIID, PAPIIID1, PAPIIID2); PAPIVa (PAPIVa, PAPIVa-g, PAPIVa-p); PAPIVb (PAPIVb, PAPIVb-g, PAPIVb-p); cytology ≤ PAPII (PAPI, PAPII, PAPIIa, PAPII-g, PAPII-p). Histologic findings were classified based on CIN classification. Colposcopic findings were classified according to the current International Federation of Cervical Pathology and Colposcopy (IFCPC) nomenclature [24]. The 2018 FIGO staging was used to classify cervical carcinomas [25]. HPV testing was performed by a cervical smear, external findings were obtained from the medical record. If external and internal cytology differed at the initial presentation, the higher-grade finding was used. To determine remission, progression, and persistence rates, patients were grouped according to findings at initial presentation and postpartum outcome. Preferably, classification was based on histologic findings. If no biopsy was taken at the time of initial presentation or if it was inconspicuous despite abnormal cytology and/or colposcopy, patients were classified according to cytologic findings at the time of initial presentation. If no biopsy was performed in patients with PAPIIID (Munich Nomenclature II) or if the biopsy was inconspicuous, the patients were assigned to the CIN 1 group. A change between CIN 1 and CIN 2 was considered persistence. Patients with PAPIII, PAPIII-p, or PAPIII-g and unperformed biopsy or inconspicuous histology were not considered in the evaluation of regression, persistence, and progression rates. Postpartum outcome was evaluated based on cytological and, if available, histological findings. Preferably, classification was based on histologic findings. If no biopsy was taken postpartum or no excision was indicated, classification was based on the cytological findings.

### Data processing and statistics

Microsoft^®^ Excel^®^ for Windows was used for data processing and IBM SPSS Statistics^®^, version 26 for Windows was used for statistical analysis. Ordinal logistic regression analysis was performed to determine the impact of delivery mode and parity on the postpartum outcome.

## Results

Of 229 patients assessed, 142 patients could be included in the further analysis. 87 patients were excluded from further analysis due to missing follow-up examinations or discontinuation of pregnancy.

### Findings at first clinical assessment

At initial presentation, ten (7.0%) patients were found to have PAPIII, 55 (38.7%) PAPIIID, 71 (50.0%) PAPIVa, four (2.8%) PAPIVb, and two (1.4%) PAPV Table 1*.* 77 (54.2%) of patients showed histologic CIN 2 + at initial presentation. Colposcopic abnormalities were detected in 130 (91.5%) patients, including minor changes in 43 (30.3%) and major changes in 85 (59.9%) cases Table 1.

**Table 1:** Patient characteristics at first presentation

No. of patients, *n*	229
No. of patients enrolled, *n* (%)	**142 (100)**
Age (years), mean (range)	31.1 (18–43)
Gravidity, median (range)	2 (1–7)
Parity, median (range)	0.5 (0–4)
BMI, mean (range)	26.7 (18.0–66.9)
Cytology (PAP), *n* (%)	
PAP III (-p/-g)	10 (7.0)
PAP IIID	55 (38.7)
PAP IIID1	7 (12.7)
PAP IIID2	14 (25.5)
PAP Iva (-p/-g)	71 (50.0)
PAP IVb (-p)	4 (2.8)
PAP V (-p)	2 (1.4)
Histology, *n* (%)	
Normal	27 (19.0)
CIN 1 (LSIL)	10 (7.0)
CIN 2 (HSIL)	11 (7.7)
CIN 3 (HSIL)	61 (43.0)
ACIS	3 (2.1)
Cervical cancer	2 (1.4)
No biopsy	28 (19.7)
Colposcopy, *n* (%)	
Normal findings	6 (4.2)
Abnormal findings	130 (91.5)
Minor changes	43 (30.3)
Major changes	85 (59.9)
Non-specific	2 (1.4)
Invasion	3 (2.1)
Others	2 (1.4)
Inadequate	1 (0.7)

*BMI *body mass index*, CIN *cervical intraepithelial neoplasia*, ACIS *adenocarcinoma in situ of the cervix*, LSIL *low-grade squamous intraepithelial lesion*, HSIL *high-grade squamous intraepithelial lesion

In 98 (69.0%) cases, the external and internal cytology findings at initial presentation were in agreement; in 29 (20.4%) patients, external cytology was of higher grade and in 14 (9.9%), it was of lower grade than internal cytology. The respective higher-grade finding was used for further evaluation. When comparing cytology and colposcopy at initial presentation (*n* = 141), concordance was found in 105 (74.5%) cases. In 19 (13.5%) patients, minor changes were found with cytological PAPIIID2 or PAPIVa and in five (3.5%) patients colposcopy was inconspicuous although cytological PAPIIID or PAPIVa was reported. Of 85 patients with colposcopic major changes at initial presentation, 80 (94.1%) had a cytologic result of the categories PAPIIID, PAPIIID2, PAPIVa, or PAPIVb. Of 75 patients with histologic CIN 2, CIN 3, or adenocarcinoma in situ (ACIS), 62 (82.7%) showed major changes, eleven (14.7%) showed minor changes, one (1.3%) had an inconspicuous colposcopy and in one patient (1.3%), suspected invasion was reported.

A T1 transformation zone was described in 105 (74.5%), T2 in 21 (14.9%), and T3 in 14 (9.9%) patients. In one case, the transformation zone was not definable because of invasive carcinoma.

Cytology and histology at initial presentation corresponded exactly in 64 (56.1%) cases, the lesion was cytologically underestimated in 11 (9.6%), and cytologically overinterpreted in 34 (29.8%). In 75 (97.4%) women with histologically confirmed CIN 2 + (*n* = 77), PAPIIID + was found.

### Postpartum therapy

Postpartum therapy of the diagnosed lesion was performed in 84 (60.4%) patients. LEEP excision was performed in 81 (58.3%) and laser vaporization in three (2.2%) patients. Therapy was performed after colposcopic and, if necessary, cytologic and histologic assessment. In 67 (82.7%) cases, the excidate showed CIN 2 + Table 2.

**Table 2:** Postpartum therapy histologic findings

Histology at therapy, *n* (%)	
Negative	12 (14.3)
CIN 1 (LSIL)	2 (2.4)
CIN 2 (HSIL)	10 (11.9)
CIN 3 (HSIL)	54 (64.3)
ACIS	1 (1.2)
Cervical cancer	2 (2.4)
Laservaporisation	3 (3.6)
Total	84 (100)

*CIN* cervical intraepithelial neoplasia, *ACIS* adenocarcinoma in situ of the cervix, *LSIL* low-grade squamous intraepithelial lesion,* HSIL* high-grade squamous intraepithelial lesion

### Remission, persistence and progression

**Fig. 1:**
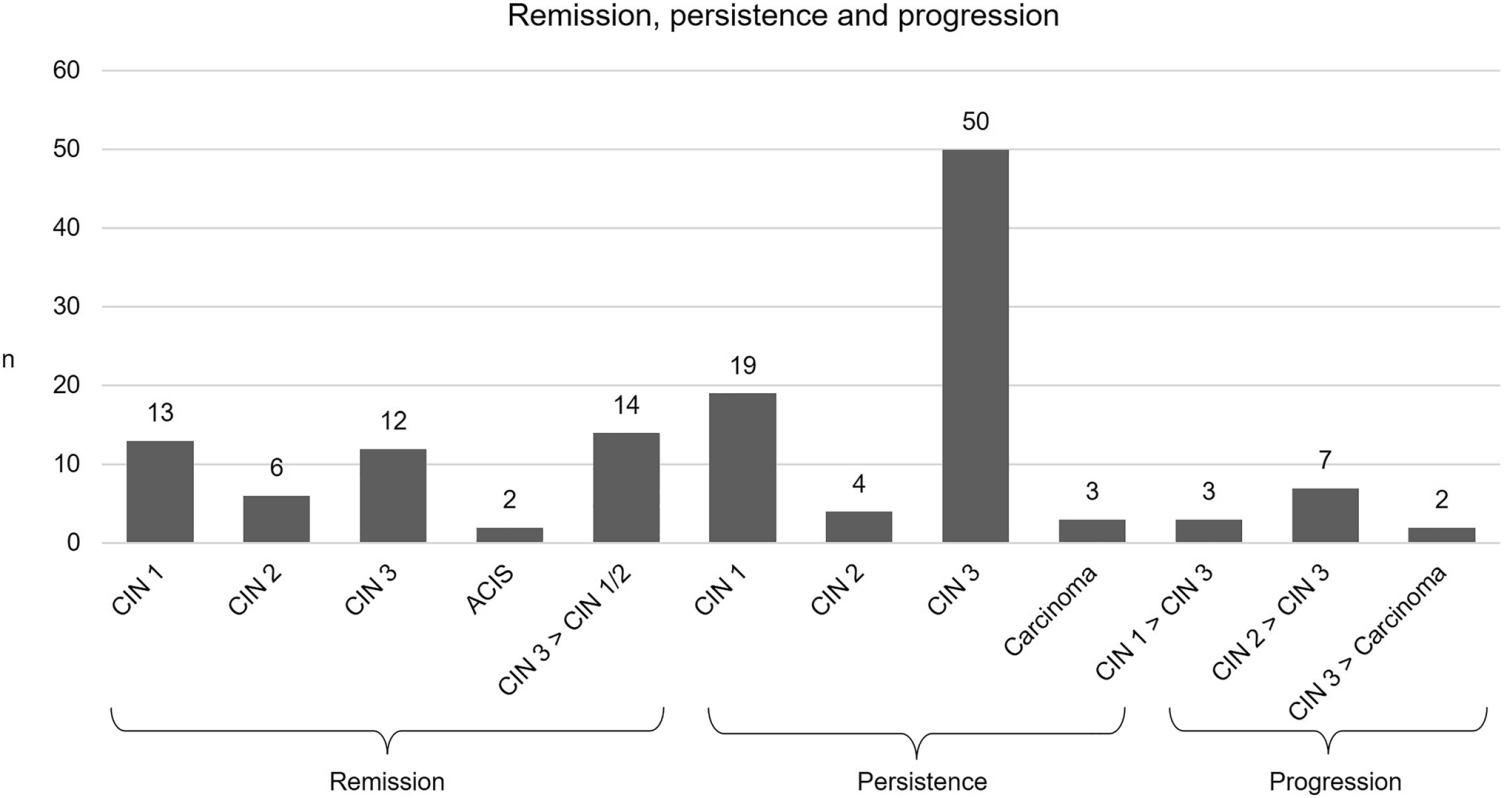
Remission, persistence and progression of dysplasia (*n* = 135). Cervical intraepithelial neoplasia (CIN), adenocarcinoma in situ of the cervix (ACIS), carcinoma of the cervix (Carcinoma)

Of 135 patients, remission of the lesion occurred in 33 (24.4%) patients, partial remission from CIN 3 to CIN 1/2 in 14 (10.4%), and persistence of the lesion in 76 (56.3%) patients. Progression was observed in twelve (8.9%) patients, including invasive carcinoma in two cases (1.5%). In patients with CIN 1 (*n* = 35), we observed remission in 13 (37.1%) cases, persistence in 19 (54.3%) and progression to CIN 3 in three (8.6%) women. In patients with CIN 2 (*n* = 17), we observed remission in six (35.3%), persistence in four (23.5%) and progression to CIN 3 in seven (41.2%) cases. A change between CIN 1 and CIN 2 occurred in one case, respectively, and was considered persistence. Of 78 patients with CIN 3, remission occurred in twelve (15.4%) cases, partial remission to CIN 1/2 occurred in 14 (17.9%) cases, in 50 (64.1%) patients, lesions persisted postpartum, and progression to invasive carcinoma was observed in two (2.6%) cases Fig. 1*.*

### Management of progression to invasive lesions

One patient presented with a first-time PAPIVa-p in the 18th week of pregnancy. Colposcopy showed major changes, histologically a CIN 3 and a high-grade vulvar intraepithelial neoplasia (VIN 3) were confirmed. Colposcopy and cytology were performed regularly during pregnancy and there was no suspicion of invasion at any time. The patient presented 9 weeks postpartum. At this time, evidence of invasive carcinoma was noted during colposcopy, cytology revealed PAPIVa-p, and histology revealed CIN 2 and CIN 3. Therapy was immediately initiated, and excision 12 weeks postpartum revealed a squamous cell carcinoma of the cervix (pT1b1, grading G3). Large conization with endocervical curettage was performed. Despite dysplasia-free resection margins, hysterectomy was recommended due to an increased risk of recurrence of the grade 3 carcinoma. After individual consultation, the patient still desired a fertility-preserving procedure. No residual primary tumor and no lymph node or distant metastases were found in the positron emission tomography–magnetic resonance imaging (PET-MRI). After the pelvic sentinel lymph node procedure, a bilateral laparoscopic pelvic lymphonodectomy was performed. A bone marrow examination was performed to detect tumor cells. There was no evidence of lymph node or distant metastasis here either. The patient was then advised to undergo a hysterectomy after the completion of family planning. To date, the patient is recurrence-free.

Another patient presented with PAPIVa (Munich Nomenclature II) in the 22nd week of pregnancy. Very difficult examination conditions existed due to severe obesity. Biopsies were taken under anesthesia during the Caesarean section, and CIN 2 was confirmed. Despite the urgent recommendation, the patient presented again only eight months postpartum. Here, a squamous cell carcinoma (pT1a1, grading G2) was diagnosed during excision. No further data are available on this patient.

### Management of invasive carcinoma at first presentation

In three patients, invasive carcinoma was diagnosed at the time of initial presentation. These patients were individually counselled and treated. In all cases, the lesion was detected by cytology, colposcopy, and histology at initial presentation.

One patient was diagnosed with FIGO stage IIA squamous cell carcinoma of the cervix (pT2a, pN0, M0, grading G3) in the 16th week of gestation. After individual consultation, a Wertheim’s procedure with sectio parva, without adnexectomy and radical pelvic and para-aortic lymphonodectomy were performed. The patient then underwent postoperative radiochemotherapy. To date, the patient is free of recurrence regarding the diagnosed and treated cervical carcinoma.

Another patient presented at 7 + 1 weeks of gestation with PAPV (Munich Nomenclature II) and suspected adenocarcinoma of the cervix. Colposcopy revealed suspicion of invasion; in the biopsies at initial presentation, the invasion could not be excluded. Based on these findings, a LEEP excision was performed in the 9th week of gestation, and a re-excision with total cervical occlusion and cerclage were performed without complications in the 13th week of gestation. There was no evidence of invasion. To quickly establish the final diagnosis and not to delay further treatment, labor was induced in the 39th week of gestation. 12 weeks postpartum, laparoscopic total hysterectomy with bilateral salpingectomy was performed. The histologic specimen showed ACIS of the cervix of 8 mm ectoendocervical length, resection margins were free or tumor. There was no evidence of an invasive tumor growth. To date, the patient is free of recurrence.

One patient was diagnosed with FIGO stage IIB squamous cell carcinoma in the 24th week of pregnancy. Colposcopy showed evidence of invasion and PAPV-p was diagnosed in cytology. Induction of fetal lung maturation was performed. After individual counseling, neoadjuvant chemotherapy with carboplatin and paclitaxel was started under close fetal monitoring. However, the tumor did not regress, so a cesarean section was performed in the 32nd week of gestation. The child showed postpartum complications like perinatal acidosis, infant respiratory distress syndrome, hyperbilirubinemia, anemia, apnea of prematurity and feeding problems which were attributed to prematurity but not to the mother's neoadjuvant chemotherapy. To date, the child has not shown any complications caused by the maternal treatment. Postpartum, the maternal tumor was found to be inoperable. The final staging revealed a squamous cell carcinoma of the cervix, grading: G2, TNM: pT3b, pN1, M1, FIGO III C. Radiochemotherapy was started. In the further course, distant metastasis became apparent and palliative chemotherapy was initiated. Due to the worsening of the general condition, the patient was transferred to an external hospital for palliative care.

### HPV

Information on the high-risk HPV status was available in 64 (45.1%) patients. Among them, 54 (84.4%) patients were high-risk HPV positive and ten (15.6%) were high-risk HPV negative. Two of the HPV-negative patients presented with PAPIII and showed a postpartum remission. One patient presented with PAPIIID, and CIN 1 was found in the postpartum biopsy. Two patients were diagnosed with CIN 2. There was postpartum progression to CIN 3 and total remission in one case, respectively. CIN 3 was diagnosed in five patients. In three cases the lesion persisted postpartum and in two cases remission occurred.

### Mode of delivery

Vaginal delivery was performed in 86 (60.6%) patients, Caesarean section in 42 (29.6%), and no data were available in 14 (9.9%) cases. In no case, the presence of CIN was the reason for performing a Caesarean section. The influence of the delivery mode on the postpartum outcome regarding remission, partial remission, persistence and progression was determined using ordinal logistic regression. Factors used were parity (primiparous or multiparous) and mode of delivery (*R*^2^ = 0.102). For vaginal delivery compared with Caesarean section, the probability of evolution towards remission appeared to be significantly increased (OR = 2.71, *p* = 0.017, 95% CI = 0.18–1.81). No significant effect could be determined for parity (OR = 1.99, *p* = 0.065, 95% CI = −  0.04–1.41).

## Discussion

The diagnosis of cervical dysplasia during pregnancy is a challenging situation for both the patient and the clinician. This study investigated whether the currently recommended management can offer the greatest possible safety for mother and child. Compared to other studies on the management of CIN during pregnancy, we were able to include data from a large number of patients in the analysis. Thus, with this work, we can contribute to further counseling and treatment of these patients. Overall, it was found that the combination of colposcopy, cytology and histology allowed a reliable diagnosis to be made during pregnancy. It is important to evaluate all the findings together with the patient in informed consent.

The evaluation of Pap smears in pregnant women may be more difficult than in non-pregnant women due to pregnancy-associated changes [6, 13]. Nevertheless, the effectiveness of Pap smears is rated as high as for non-pregnant women [14]. Colposcopy, cytology and histology should be performed and evaluated by experienced examiners. The short time interval between external and internal cytology at initial presentation may result in decreased sensitivity and a higher rate of false-negative findings [26]. Therefore, it is always important to consider both findings. A high rate of inconspicuous biopsies at initial presentation in this study could be explained by increased caution during specimen collection; in addition, endocervical curettage was never performed. Only in one of 142 patients (0.7%) the colposcopy was inadequate, which indicates the good evaluability of the cervix during pregnancy. During pregnancy, the squamocolumnar junction is usually well visualizable [12, 15]. A high rate of T1 and low rate of T3 transformation zones can also be explained by a young patient population.

Overall, of 135 patients, remission of the respective lesion was observed in 24.4%, partial remission from CIN 3 to CIN 1 and CIN 2 in 10.4%, persistence of the lesion in 56.3% and progression in 8.9%, including progression to invasive carcinoma in 1.5%. Remission, persistence and progression rates of previous publications are summarized in Tables 3 and 4*.* The patients presented by Henes et al. in 2013 are also part of the current evaluation [20]. Due to the higher number of cases, the figures in the current study vary. In one case (1.5%), invasive cervical carcinoma occurred, but treatment was significantly delayed due to delayed postpartum presentation [20] Table 3. Because CIN 1, CIN 2, and CIN 3 lesions differ in terms of their regression pattern, we evaluated the evolution of each lesion individually. In patients with CIN 1 or PAPIIID/PAPIIID1 in pregnancy, we observed remission in 37.1% (*n* = 13), persistence in 54.3% (*n* = 19) and progression to CIN 3 in 8.6% (*n* = 3). Previous publications report varying remission rates of 35.9–69%, progression to invasive carcinoma is not described [27–29] Table 3*.* In patients with CIN 2 or PAPIIID2, we observed remission in 35.3% (*n* = 6), persistence in 23.5% (*n* = 4) and progression to CIN 3 in 41.2% (*n* = 7). Hong et al. report a lower rate of total remission and progression [30]. Yost et al. report more remission and partial remission and less progression. In this study, the patient population was younger than ours [31] Table 3*.* In patients with CIN 3, remission of the lesion was observed in 15.4%, partial remission to CIN 1 or CIN 2 in 17.9%, persistence of the lesion postpartum in 64.1%, and progression to invasive carcinoma in 2.6%. Microinvasive squamous cell carcinoma (pT1a1) was diagnosed in one case and macroinvasive squamous cell carcinoma (pT1b1) in another. The literature provides widely varying data regarding regression, persistence, and progression of CIN 3 lesions. Hong et al. report similar persistence and progression rates and more total remission. Squamous cell carcinoma was found in stage pT1a1 in two cases and stage pT1b1 in one case [30]. Paraskevaidis et al. report more remission and partial remission and less persistence [27]. In contrast, Kaplan et al. did not observe remission of HSIL in any case, the lesions persisted in most cases and progression to microinvasive carcinoma occurred in 10.7%. The study population here also was younger than ours [28]. There are also reports of very high remission rates for HSIL in pregnancy. For example, Vlahos et al. observed remission or partial remission in over 60% [32], Fader et al. observed total remission in 53% and partial remission in 16% [18]. Similarly, Yost et al. describe a remission and partial remission for CIN 3 of 70% [31] Table 4*.* The course of CIN during pregnancy has been reported differently. On the one hand, this may be due to differences in the study populations regarding size and age as well as diagnostics performed during pregnancy, and on the other hand, it must be noted that all studies are retrospective analyses with rather small cohorts. Despite the varying results of different studies, it can be stated that progression to invasive carcinoma of CIN in pregnancy is rare. A wait-and-see approach can therefore be justified. Postpartum follow-up and, if necessary, treatment of the lesion are essential.

**Table 3:** Comparison of previously published remission, persistence and progression rates of all lesions, CIN 1 and CIN 2 [20, 27–31]

	Total		CIN 1				CIN 2		
	Current study	Henes et al. [20]	Current study	Paraskevaidis et al. [27]	Kaplan et al. [28]	Serati et al. [29]	Current study	Hong et al. [30]	Yost et al. [31]
Remission	24.4	40.0	37.1	35.9	62	69	35.3	27.7	68
Partial remission	10.4	4.6					23.5	17.0	
Persistence	56.3	26.2	54.3	59.0	32	16.6		19.1	25
Progression	8.9	1.5	8.6 (CIN 3)	5.1 (CIN 2/3)	6 (CIN 2/3)	14.3 (CIN 2/3)	41.2	36.2	7

Cervical intraepithelial neoplasia (CIN)

**Table 4:** Comparison of previously published remission, persistence and progression rates of CIN 3 and CIN 2/3 (HSIL) [18, 27, 28, 30–32]

	CIN 3			CIN 2/3			
	Current study %	Hong et al. [30] %	Yost et al. [31] %	Paraskevaidis et al. [27] %	Kaplan et al. [28] %	Vlahos et al. [32] %	Fader et al. [18] %
Remission	15.4	26.5	70	25.0	0.0	61.5	53
Partial remission	17.9	7.1		23.1	0.0		16
Persistence	64.1	63.7	30	50.0	89.3	38.5	31
Progression	2.6	2.7	0	1.9	10.7	0.0	0

*CIN* cervical intraepithelial neoplasia, *HSIL* high-grade squamous intraepithelial lesion

In one case, progression to macro invasive squamous cell carcinoma was observed. In this case, the progression cannot be explained by delayed postpartum presentation or missed follow-up examinations. There was no colposcopic, cytologic, or histologic suspicion of invasion in the patient at any time during pregnancy. 9 weeks postpartum, colposcopic signs of invasion were noted, and cytology and histology revealed PAPIVa-p with CIN 2 and CIN 3. Therapy was immediately initiated. A squamous cell carcinoma of the cervix, pT1b1, G3 was diagnosed in the excidate. To assess whether, knowing the squamous cell carcinoma diagnosed later, the evaluation of the biopsies and the images of the colposcopic examinations during pregnancy would have been different, they were re-evaluated in this study. Again, there was no suspicion of malignancy. The cause of progression can only be speculated. Immunosuppressed women are at increased risk for developing CIN 3 + [33]. Combined CIN 3 + and VIN 3 + lesions are more common in HPV-positive and immunosuppressed women [34]. However, no previous disease was reported by the patient, especially no immunosuppression. The presence of a highly malignant lesion with grading G3 may be another factor for rapid progression. Diagnosis could be more difficult in the presence of carcinoma growing from the endocervix. Increased error rates have been described for the detection of CIN 3 + lesions in women with T3, unremarkable cytology, and concurrent high-risk HPV positivity. The risk of not detecting an endocervical located lesion increases [35]. In addition, endocervical curettage should not be performed in pregnant women [12]. In the patient described, a T1 transformation zone was always present, so there was no indication for curettage even postpartum. The colposcopic suspicion of invasion described by the examiners at postpartum presentation could not be confirmed despite a colposcopy-guided biopsy and Pap smear. Due to the retrospective nature of the study, it is difficult to elaborate on the reasons for the rapid progression. However, this case makes it particularly clear that postpartum presentation in time is essential, especially in the case of CIN 3 diagnosed during pregnancy. Any necessary therapy should be performed without delay.

In another case, progression to invasive carcinoma was observed. In this case (squamous cell carcinoma of the cervix, pTa1, grading: G2), despite the urgent recommendation, the patient presented for follow-up only eight months postpartum. This case also highlights the importance of early postpartum presentation and treatment of high-grade dysplasia. In this case, the progression can be explained by the failure to attend check-ups and the resulting delay in treatment.

In three cases, an invasive carcinoma was suspected at initial presentation. In all these cases, the lesion could be detected in all three examination methods. Invasive carcinoma in pregnancy requires acute treatment and a waiting approach cannot be justified in all cases. Examinations during pregnancy should be performed by experienced clinicians [12, 18]. After histological confirmation of the diagnosis, the patient should be discussed on a tumor board. Further therapy is always individual therapy. The prognosis of pregnant women with gynecologic carcinomas does not appear to differ from that of non-pregnant women [6].

In one case, a FIGO stage IIA squamous cell carcinoma of the cervix was diagnosed at the 16th week of gestation. The current German guideline recommends that if cervical carcinoma is diagnosed at FIGO stage IB and IIA before the 16th week of gestation, termination of pregnancy should be performed and therapy should be started [36]. This approach was also followed in the affected patient. If diagnosed at a later stage, waiting for fetal lung maturity may also be considered, with potential neoadjuvant chemotherapy starting in the second trimester [36]. In one case, a cervical squamous cell carcinoma was diagnosed in the 24th week of gestation. Initially, FIGO stage IIB was assumed. Diagnosis of such advanced carcinoma in pregnancy is rare [21]. Due to the diagnosis at the end of the second trimester, the patient and the attending physicians decided to start neoadjuvant chemotherapy and to wait for fetal lung maturity after the presentation to the tumor board.

In one case, suspicion of invasion could not be excluded in a patient with ACIS in the 8th week of pregnancy. Therefore, LEEP excision during pregnancy was recommended. In the case of ACIS, the indication for excision should be made generously because it is often not possible to exclude invasion with certainty [12]. In the case of microinvasive carcinoma in pregnancy, excision is recommended if the diagnosis is made until the 14th week of gestation, despite the high risks such as miscarriage, hemorrhage, and prematurity [6, 21]. In this case, the indication for excision during pregnancy can be justified because invasion could not be excluded and the cytologic and colposcopic findings were suspicious of invasion. The procedures were performed without complications and a healthy child was born.

The treatment of pregnant women with invasive carcinomas is a major challenge for all parties involved. It is important that decisions regarding therapy and pregnancy are always made by a specialized and interdisciplinary team. The diagnosis of cervical carcinoma in pregnancy is rare. The management of an invasive carcinoma is always an individual decision, considering the current guidelines but also the wishes and needs of the patients concerned [36, 37].

High-risk HPV status was only available for 45.1% of patients. Due to the new screening for cervical cancer in Germany which started in 2020, we expect more information on the high-risk HPV status in the future, especially in patients aged 35 and older [38]. High-risk HPV-negative patients could also have low-grade dysplasia caused by low-risk HPV and a large time interval between HPV sampling and the colposcopic examination could also be an explanation.

In this study, the presence of cervical dysplasia has never been the reason for performing a Caesarean section. The influence of the mode of delivery on the likelihood of postpartum remission is controversial. Some authors report increased rates of regression of CIN in women after vaginal delivery [27, 39], whereas other studies demonstrate no influence [30, 31, 40, 41]. The influence of the mode of delivery on postpartum outcome was assessed using ordinal logistic regression. A significantly increased probability of evolution toward remission was found in women with vaginal delivery. However, only 10.2% of the variance could be explained by this model. In contrast, Gomez et al. report a significant effect of parity on the likelihood of remission, whereas they were unable to determine an effect for delivery mode [41]. In our study, the determined influence on the course of dysplasia is small, there seem to be further influencing factors, which could not be investigated and evaluated in this work. The results cannot be transferred to the prognosis of an affected patient. Furthermore, due to the small group sizes, statistical bias cannot be excluded. It is important for the clinical practice that there is no indication for delivery by caesarean section due to a CIN; no negative effects of a vaginal delivery could be determined. The presence of invasive carcinoma, however, is a contraindication to vaginal delivery [21].

## Conclusions

In summary, not treating diagnosed CIN during pregnancy seems to be safe for mother and child. Close monitoring by cytology, colposcopy and histology should be performed by experienced examiners during pregnancy. In order not to delay treatment, especially of high-grade lesions, a postpartum presentation around 6 weeks after delivery is essential. Therapy in case of diagnosed CIN does not seem necessary during pregnancy. In the case of CIN, spontaneous delivery is safe, there is no indication for a Caesarean section.
